# Delayed Diagnosis of an Atypical Pneumonia Resembling a Solitary Pulmonary Nodule

**DOI:** 10.7759/cureus.19456

**Published:** 2021-11-10

**Authors:** Rafael Garcia-Carretero, Oscar Vazquez-Gomez, Belen Rodriguez-Maya, Franciso Garcia-Garcia

**Affiliations:** 1 Department of Internal Medicine, Hospital Universitario de Mostoles, Mostoles, ESP

**Keywords:** percutaneous nodule biopsy, chest x-ray, solitary pulmonary nodule, atypical pneumonia, covid-19

## Abstract

Atypical pneumonia shows clinical features that are different from those of typical pneumonia, and it can mimic other entities. We report the case of a 42-year-old male with a solitary pulmonary nodule found in an X-ray for a preoperative evaluation. Our patient was asymptomatic, and a pulmonary neoplasm was the first diagnostic suspicion. The round-shaped nodule seen in the X-ray turned out to be a linear ground glass opacity in a thoracic CT scan. Viral pneumonia due to SARS-CoV-2 was diagnosed. We emphasize here the educational value of this case report. We do not report a new radiological finding because lung nodules resembling neoplasms have already been reported in the medical literature. However, some clinical features of COVID-19 are relatively new and can mimic other entities, and the results of some investigations and clinicians’ interpretations of them can be misleading. Atypical radiological findings make it necessary to widen the spectrum of alternative diagnoses.

## Introduction

Some pulmonary diseases may be elusive when diagnosed via plain chest X-ray as they can mimic other entities. Atypical pneumonia, caused by atypical bacteria (such as *Mycoplasma pneumoniae*), viruses, or fungi, is uncommon but tends to have subacute courses or milder symptoms than typical pneumonia. Atypical pneumonia is often community acquired and mild to moderate, although signs and symptoms vary. Its onset is gradual and can be accompanied by headache, dry cough, malaise, or even low-grade fever. Dry cough is often a common complaint. Dyspnea is less common when compared with pneumonia caused by other pathogens such as *Streptococcus pneumoniae*. There is no pathognomonic sign, and even chest auscultation can be normal. Therefore, atypical pneumonia can be asymptomatic, and a chest X-ray is usually used to diagnose ground glass opacities, although reticular, nodular, or patchy opacities can be also seen. Since its course is mild, most patients recover with no antibiotic therapy. However, atypical pneumonia can also resemble a pulmonary neoplasm. In such cases, a systematic diagnostic workup should be performed for the final diagnosis. Given the relatively recent onset of the COVID-19 pandemic, some of its clinical features are not yet well understood, especially in asymptomatic patients. Some clinical and radiological features of COVID-19 are relatively new and can mimic other entities. Hence, clinicians should be aware of symptoms and signs that can be misleading.

## Case presentation

**Figure 1:**
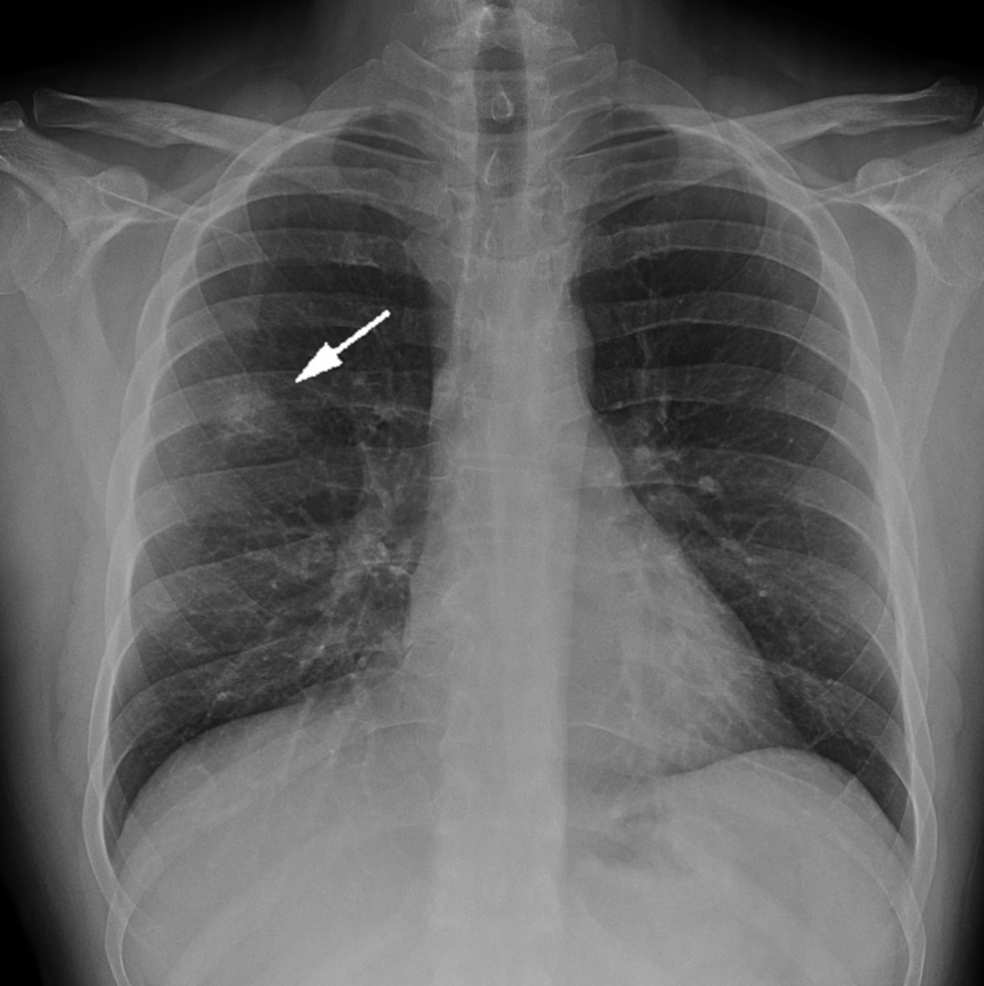
Chest X-ray A plain chest X-ray showing a solitary pulmonary nodule 2 cm in diameter (arrow) in the right mid-lung

In September 2020, a 42-year-old male was referred to our Department of Internal Medicine because of a finding in a chest X-ray. The patient was healthy with no previous hospitalizations and worked as a nurse at our institution. He was not taking any medications, had no smoking history, and was presenting no malignancy-related symptoms (fatigue, unintended weight loss, or changes in bowel habits). He had seen an anesthesiologist before being seen by a surgeon due to an inguinal hernia, a minor condition. The anesthesiologist noticed the pulmonary lesion. A chest X-ray showed a solitary pulmonary nodule in the right mid-lung that was 2 cm in diameter (Figure 1). The patient was asymptomatic, as mentioned above, and had a previous chest X-ray that was normal.

In a physical examination, his temperature was 36.7°C, blood pressure was 138/78 mm Hg, heart rate was 76 beats per minute, and oxygen saturation was 98% in room air. In auscultation, heart and lung sounds were normal. Both oropharyngeal and abdominal examinations were normal, and he had no periodontal disease. The patient was admitted to the hospital for further investigation.

Blood tests were normal, with a white blood cell count of 9,430 leucocytes/mm^3^ with 63% neutrophils, hemoglobin level of 153 g/L, and platelet count of 205 × 10^9^/L. C-reactive protein was 83 mg/dL (normal range: <5 mg/dL). A reverse-transcription polymerase chain reaction (RT-PCR) test was negative on hospitalization day 1 (Table 1). As mentioned, a chest X-ray showed a solitary pulmonary nodule in the right upper lobe.

**Table 1:** Summary of laboratory testing during hospital stay RT-PCR: reverse-transcription polymerase chain reaction

Laboratory parameters	Day 1	Day 3	Reference
White blood cell count	9,430 leucocytes/mm^3^	10,100 leucocytes/mm^3^	6,000–14,000 leucocytes/mm^3^
Neutrophils	63%	69%	40%–60%
Lymphocytes	29%	20%	20%–40%
Hemoglobin	153 g/L	150 g/L	140–180 g/L
Platelet count	205 × 10^9^/L	180 × 10^9^/L	150–400 × 10^9^/L
C-reactive protein	83 mg/dL	79 mg/dL	<5 mg/dL
RT-PCR	Negative	Positive	

**Figure 2:**
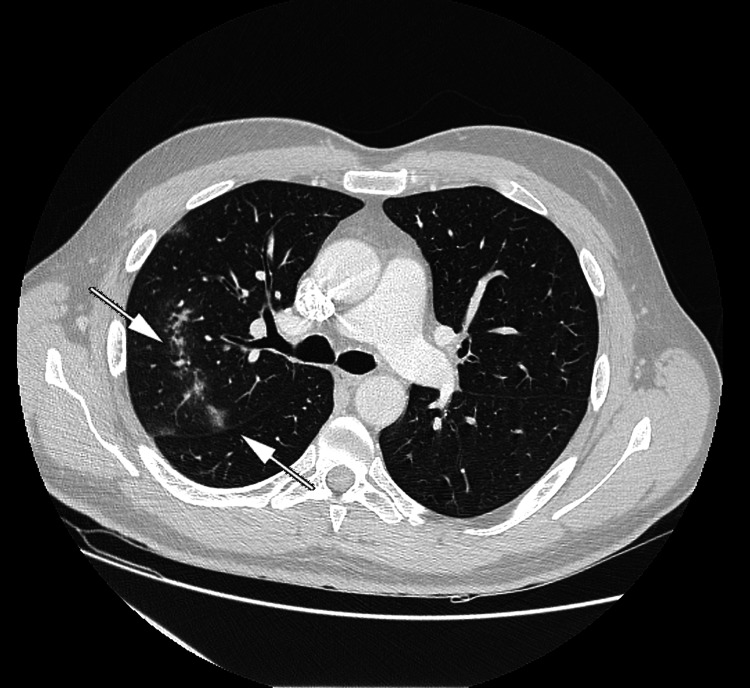
Thoracic CT scan Slide of a thoracic CT scan revealing linear-shaped ground glass opacities in a transversal plane in the upper right lobe (arrows), which resembled round-shaped opacities in the frontal plane of a plain chest X-ray

Our patient presented with a solitary pulmonary nodule, which raised the suspicion of a primary lung tumor or metastasis of unknown origin. For further radiological characterization and assessment of the pulmonary node, the patient underwent a thoracic CT scan, which revealed ground glass opacities that suggested either inflammatory or infectious conditions (Figure 2), similar to atypical pneumonia. A second RT-PCR was performed on hospitalization day 3, and the result was positive. Based on these data, we established a diagnosis of COVID-19 pneumonia in an asymptomatic patient. The first test for the qualitative detection of IgG against SARS-CoV-2 (a chemiluminescent microparticle immunoassay) was negative on hospitalization day 3.

On day 5, the examination was normal, the patient’s oxygen saturation was 98%-99% in room air, and laboratory blood tests were normal, so we discharged the patient with no treatment, but he was attended in a follow-up in our outpatient clinic with a new chest X-ray and new laboratory blood tests on day 10 after discharge. The chest X-ray showed complete resolution of pneumonia, and laboratory blood tests were normal, with C-reactive protein < 5 mg/dL and a positive test for IgG against SARS-CoV-2.

## Discussion

SARS-CoV-2 was first identified in 2019, and the disease it causes was named COVID-19 by the WHO in 2020 [1,2]⁠. Its symptoms include fever, dry cough, myalgia, and dyspnea [3]⁠, but an asymptomatic course is not uncommon. RT-PCR is the gold standard for diagnosis, and several imaging techniques, such as chest X-ray, thoracic CT scan, and ultrasound, are useful for the diagnosis and assessment of COVID-19 pneumonia [4]⁠.

Bilateral multifocal ground glass opacities in the mid- or lower lobes are the most common initial findings in chest X-rays [5,6]⁠. However, during the early stages of the disease, chest X-rays may have limited usefulness due to the possibility of false-negative results. Hence, thoracic CT or ultrasound can be used in early diagnosis, even if an initial RT-PCR is negative. COVID-19 pneumonia findings are nonspecific. Any viral, fungal, or atypical bacterial infection can mimic ground glass lung opacities [7]⁠. In such cases, a complete laboratory blood panel, including blood cultures, serology, and nasopharyngeal smears for RT-PCR, is required [4]⁠.

In our patient, COVID-19 pneumonia resembled a solitary pulmonary nodule. Given that the patient presented with no further symptoms, our first suspicion was a pulmonary neoplasm, either primary or metastatic. The round opacity misled us, and the first negative RT-PCR did not correct the first erroneous assessment. A thoracic CT scan demonstrated that a linear shape in a transverse plane can be seen as a rounded nodule in a frontal plane. That is, both the shape of the opacity and the negative RT-PCR were misleading in establishing the final diagnosis.

Radiological findings are used to determine the stage and severity of COVID-19 pneumonia. Asymptomatic individuals can present with unique, isolated, peripheral lesions [4-6]⁠, and although isolated nodules are common in viral pneumonia, several cases have been reported where small, solitary pulmonary nodules appear as the initial presentation of COVID-19 pneumonia [8-10]⁠. The opposite can also occur, however. A case from Spain was recently published where bilateral COVID-19 pneumonia was diagnosed based on chest X-ray results. A thoracic CT scan demonstrated metastatic pulmonary nodules and lytic bone lesions [11]⁠. This case report should make clinicians aware of differential diagnoses other than COVID-19, as its clinical features may resemble those of other entities.

In our case report, a chest X-ray was demonstrated to be less sensitive than thoracic CT for detecting lung disease. We like to emphasize that RT-PCR, not thoracic CT, is the current gold standard for diagnosing COVID-19, although the techniques are correlated [5]⁠.

Regardless of lung involvement, the diagnosis of COVID-19 is based on clinical features (symptoms and physical signs) and/or laboratory and imaging abnormalities, and it is confirmed with RT-PCR [3]⁠. A negative RT-PCR result should be interpreted cautiously if clinical features and radiological findings raise reasonable suspicion. In such cases, several RT-PCR tests should be performed, from either nasopharyngeal or bronchoalveolar samples. Indeed, a thoracic CT scan shows a similar performance when COVID-19 with lung involvement is diagnosed, even when RT-PCR produces negative results [5]. It is worth noting that a recent publication reported that up to 20% of individuals with COVID-19 remain asymptomatic [12]⁠, as in our patient. Ultimately, a positive RT-PCR result, the finding of pneumonia in a thoracic CT scan, and a positive IgG against SARS-CoV-2 helped us establish a final diagnosis of COVID-19 pneumonia that otherwise would have been misdiagnosed at the beginning of the hospitalization.

Our manuscript does not propose or report a new radiological finding because, as stated within the manuscript, lung nodules resembling neoplasms have already been reported in the medical literature. With our work, we highlight that the results of some investigations and clinicians’ interpretations of them can be misleading. This is the educative value of our manuscript, as also seen in a recent paper [13]⁠. We agree that the radiological findings mentioned were commonly found in the COVID-19 pandemic. However, as mentioned earlier, we did not consider this the learning point or the focus of the manuscript. We did not intend to highlight the linear pattern of ground glass opacities in itself but rather the way in which it resembled a suspicious pulmonary nodule and how a suspicion of malignancy may initiate a workup on a neoplasm.

Moreover, during the current pandemic, imaging techniques have played a relevant role in supporting the diagnosis of COVID-19, as the lung is the main organ involved. Both X-rays and CT studies can identify pulmonary involvement or complications, and both can suggest different diagnoses. However, the most common radiological findings in infections caused by SARS-CoV-2 are ground glass opacities, typically bilateral, peripheral, and located in the lower fields [14]⁠. This finding may progress to a diffuse disease. However, atypical or uncommon findings, such as lung nodules, miliary patterns, pleural effusion, or cavitation, are reported in only 3% of patients [15]⁠.

Having said that, we stress that our patient showed an indeterminate or atypical appearance of radiological signs for COVID-19 pneumonia in both X-ray and CT studies, conforming to the medical literature [14,16-18]⁠. It is worth noting a recent paper discussing atypical radiological findings that make it necessary to widen the spectrum of alternative diagnoses [19]⁠.

## Conclusions

Chest X-ray should be the first diagnostic approach for imaging, but it may show nonspecific patterns that could imply other entities, such as other infectious pulmonary diseases or neoplasms. Thoracic CT scan may help establish a diagnosis of COVID-19 pneumonia, being more sensitive than a chest X-ray, but it should not be the imaging technique of first resort due to the exposure to ionizing radiation and the expense of the procedure. The initial results of a negative RT-PCR test should be interpreted cautiously if clinical features and radiological findings suggest COVID-19. In such cases, multiple tests should be performed, even from bronchoalveolar lavage if necessary. For a relatively new disease such as COVID-19, clinicians should be aware of certain manifestations, as not all of the clinical features will have been described yet. In some scenarios, it remains unclear which manifestations are typical and which are not.
